# Integrating evolutionary theory into a framework for the mechanistic evaluation of candidate anti-aging interventions

**DOI:** 10.3389/fragi.2026.1800288

**Published:** 2026-06-15

**Authors:** Yusuf Aggour, Roberto Salguero-Gómez

**Affiliations:** 1 Pembroke College, Oxford, United Kingdom; 2 Department of Biology, Life and Mind Building, Oxford, United Kingdom

**Keywords:** caloric restriction, disposable soma theory, gene expression profiling, geroprotectors, hyperfunction theory, rapamycin

## Abstract

Despite decades of research into the molecular hallmarks of aging, geroscience lacks a unifying framework that both coherently integrates evolutionary accounts of aging and is directly useful for intervention assessment. Here, we propose the ‘Aging Onion’, a layered, two-axis framework developed to help interpret the diversity of phenotypes associated with aging, and informed by the mechanistic emphases foregrounded by the Disposable Soma and Hyperfunction traditions. In this framework, persistent activity of growth and developmental programmes, alongside insufficient somatic maintenance, define two broad biological axes through which we attempt to interpret age-related degeneration, and intervention responses. To examine the utility of this framework, we focus on nutrient sensing as an initial benchmark domain. Within this space, caloric restriction is a conserved reference intervention, while rapamycin provides a narrower pharmacological comparator acting primarily through TOR-centred signalling. We ask whether the proposed axes of the Aging Onion can help ground the mechanistic divergence between these interventions in aging-relevant biology and thereby interpret their differing efficacies. We conclude by outlining future directions for testing whether this framework can be operationalised in broader intervention assessment, and for determining whether its layered logic generalises beyond nutrient sensing to other domains of aging biology, including population-genetics approaches.

## 1 Introduction

Aging is the dominant risk factor for nearly all chronic human diseases (Niccoli and Partridge, 2012). Here, we refer to aging as both the passage of time and the functional decline that can accompany it. While the latter is most commonly termed senescence (Gilbert, 2000), this word has cell-specific connotations and can be inconsistently applied in biomedical contexts. We therefore use ‘aging’ throughout. By 2050, over one in six people worldwide are projected to be over the age of 65 (United Nations, 2022). All else being equal, the resulting rise in age-related disease will worsen existing strain on social, economic, and healthcare systems (WHO, 2015; Vos et al., 2020). Aging is significant not only because it limits lifespan but more importantly because it constrains healthspan – the period of life spent in good health, free from chronic disease and functional decline (Crimmins, 2015).

In the late 20th century, key medical advances were made in the investigation of the pathophysiology of age-related disease (Fries, 1980). Examples include the mechanistic underpinnings of cardiovascular disease and cancer (Weinberg, 1989; Dzau et al., 2006). In parallel, biology more broadly investigated conserved mechanisms of deterioration across the tree of life (Partridge and Gems, 2002). The different investigatory lenses of medicine and biology map onto the concepts of proximate and ultimate explanations of aging, respectively. Proximate explanations address the *how* – the immediate mechanisms precipitating a biological event. Ultimate explanations address the evolutionary *why* - asking why a trait evolves and how it persists through natural selection. Our thesis here is that biogerontology has much to gain by integrating both types of explanation.

In medicine, proximate explanations of phenomena are valued for their mechanistic clarity and actionable translational potential. However, the diverse pathophysiologies of age-related diseases do not readily converge on a common point of vulnerability. Recognition of this limitation (Ladislas, 2000) prompted investigation into the shared proximate mechanisms of aging across different tissues and pathologies, leading to the ever-expanding list of ‘hallmarks of aging’ (López-Otín et al., 2023). The hallmarks have become a highly influential framework for organising age-related biology and, increasingly, candidate gerotherapeutic interventions (Lifespan Research Institute, 2026). However, hallmark-based organisation does not by itself provide a full account of aging’s broader causal structure, nor does it specify how distinct mechanistic targets relate to deeper life-history trade-offs. This issue is as much practical as it is conceptual. As the intervention landscape grows denser, and multiple therapeutic strategies are being mapped onto different hallmark domains, a higher-order organising level becomes useful for asking whether apparently diverse age-related phenotypes can be situated along more general causal axes, with shared therapeutic vulnerabilities.

In contrast to the proximate lens which has produced the hallmarks, ultimate explanations of aging lie upstream and offer the prospect of identifying a conserved regulatory network that evolution has already exploited to extend or constrain lifespan across species. If natural selection can act on this network to modulate longevity, then it may also point towards vulnerabilities relevant to therapeutic intervention (Kirkwood, 2005). Many ultimate explanations also recognise that this network may be modulated within organismal lifetime. Caloric restriction (CR) exemplifies this principle. CR is an evolved response to resource scarcity whose broadly conserved (Speakman and Mitchell, 2011; Deere et al., 2023) anti-aging effect across taxa suggests modulation of central mechanisms linked to aging regulation. However, we note that the practical implementation of CR in humans is likely challenging owing to behavioural, social, and physiological constraints. This translational barrier has motivated efforts to develop CR mimetics (Speakman and Mitchell, 2011), *i.e.*, pharmacological interventions that reproduce its benefits. The most extensively studied candidate is rapamycin (Lamming et al., 2013), an mTOR inhibitor, yet its effects fall short of CR in many models (Unnikrishnan et al., 2020). If CR is an evolved, adaptive modulation of the regulatory network identified by ultimate theories of aging, it is a benchmark against which mechanistic models of this network can be tested. A credible model ought to explain both the mechanistic basis of CR and the shortcomings of current mimetics.

We therefore need a conceptual framework that is evolutionarily grounded and mechanistically informed; capable of explaining both the CR response and the differing efficacies of its mimetics. Here, we develop a pathology- and intervention-facing synthesis of two broad biological vulnerabilities recurrently foregrounded in the evolutionary aging literature. We use this synthesis to ask whether age-related pathology and intervention response can be organised around these interacting biological axes. To this end, we integrate the mechanistic emphases of two prominent evolutionary traditions – the Hyperfunction theory (Gems, 2022) and the Disposable Soma theory (Kirkwood, 2005) - into a unified framework that seeks to:

- provide a higher-order lens for interpreting diverse age-related phenotypes and pathologies
- help explain the mechanistic basis of adaptive anti-aging responses such as caloric restriction, and
- generate a research agenda for understanding why candidate mimetics and related interventions may diverge in efficacy

Recent work, including that of Le Maître and colleagues (Lemaître et al., 2024), has already advanced the broader integration of the disposable-soma and developmental accounts of aging, including Hyperfunction theory, within an evolutionary framework. Rather than proposing another general hierarchy for the evolution of aging, we independently develop this synthesis through phenotype- and intervention-facing logic. Specifically, we ask whether two broad axes of life-limiting biology may jointly shape age-related phenotypes, pathologies, and the efficacy of anti-aging interventions. The first is the pathological persistence of growth and developmental pathways, foregrounded most strongly by Hyperfunction theory. The second is suboptimal somatic maintenance and repair, articulated most fully within the Disposable Soma tradition but not exclusive to it, since developmental accounts can also generate late-life maintenance failure through different evolutionary logics. We therefore treat both axes as evolutionarily informed but proximate biological categories. For maintenance insufficiency in particular, our aim is to operationalise this axis largely independently of the differing evolutionary explanations that may give rise to it.

**FIGURE 1:**
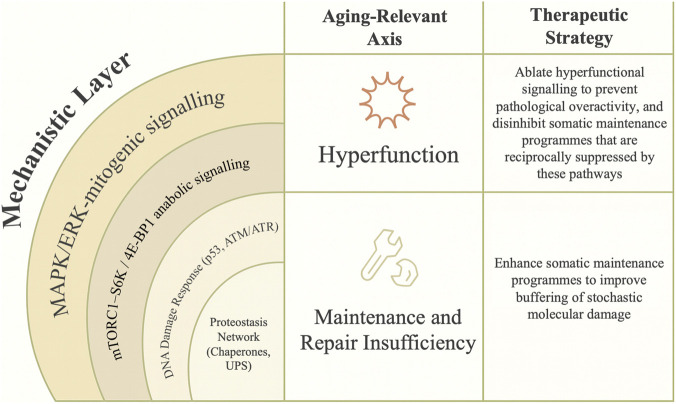
The Aging Onion: a layered, dual-axis model for interpreting age-related pathology and intervention efficacy. The model organises age-related biology around two broad, evolutionarily informed but proximate axes: persistent activity of growth-promoting pathways beyond their adaptive window (*hyperfunction*) and insufficient effective somatic maintenance and repair (*maintenance-insufficiency*). Each concentric layer represents a more specific mechanistic process through which one of these axes may be instantiated. The ‘Aging-Relevant Axis’ column summarises how each layer contributes to organismal decline through one of two broad biological axes: pathological overactivity (hyperfunction) or through insufficient somatic maintenance (maintenance and repair function, MRF, failure). Corresponding therapeutic strategies map onto each type of layer: suppression of hyperfunctional signalling to prevent pathological overactivity, and upregulation of maintenance programmes to buffer stochastic damage. The “onion” metaphor emphasises that interventions can act on single or multiple layers, and that more comprehensive strategies ought to engage both hyperfunction and maintenance layers. The layers depicted are schematic rather than quantitative. Their number, order, and relative thickness are not intended to indicate fixed biological importance, but to illustrate how multiple life-limiting processes may coexist and vary in relative salience across contexts.

Our synthesis, termed the Aging Onion (Figure 1), provides a layered view of aging biology which operationalises the idea that aging can be understood as a series of stacked, life-limiting processes (Gems, 2022; de Magalhães, 2012). We use the Aging Onion as a framework for asking whether a joint hyperfunction-maintenance lens can help explain both the aging phenotype and divergence in anti-aging intervention efficacy. In the present paper, we use nutrient sensing as an initial benchmark domain through which to explore the Aging Onion’s utility, focusing on the contrast between CR and rapamycin as a proof-of-principle case. We do not treat this domain as exhaustive; rather, we use it as a window onto a broader research agenda, which future work will need to test across other mechanistic and pathological contexts, and the broader aging literature.

## 2 Integrating evolutionary theories of aging: The “Aging Onion” framework

In this section, we first summarise the prominent evolutionary thinking regarding aging before introducing the narrower synthesis developed in this paper. Evolutionary theories of aging seek to ultimately explain the progressive functional decline of organisms that leads to increasing risk of disease, loss of vitality, and eventual death (Kirkwood, 2005). These theories must not only account for the aging phenotype, but its occurrence in the first place. Early group-selection explanations suggested that an aging programme exists to secure a turnover of generations (Weismann et al., 1891). However, patterns of age-related mortality in wild populations suggest that organisms may succumb to extrinsic mortality before age-related deterioration can be identified (Lack, 1954). Most modern evolutionary accounts therefore treat aging as a non-adaptive by-product of declining selection with age. In this view, aging occurs in a “selective shadow” (Medawar, 1953); its deleterious effects arise only late in life, at which point individuals do not (at least directly – see the “grandmother hypothesis” (Hawkes, 2004)) contribute to the performance of the population (Medawar, 1953). Under this view, the phenotype of aging is not directly programmed by evolution. More recent programmatic hypotheses have nevertheless been proposed, including pathogen-driven aging (Lidsky and Andino, 2022) in which strong selection is argued to favour conserved aging mechanisms that limit lifespan to reduce the burden and transmission of chronic infection within structured populations (Lidsky and Andino, 2022). However, these accounts provide much less empirically developed descriptions of conserved physiological decline compared to theories that treat aging as a detrimental byproduct of evolution under declining selection.

The starting point for most evolutionary accounts of aging is the declining force of selection with age. Classical evolutionary-genetic models specify two main ways in which this declining selection can generate aging. Under mutation accumulation (MA) theory, deleterious alleles with predominantly late-life effects can persist because selection against them is weak (Mc Auley, 2025). Under antagonistic pleiotropy (Mc Auley, 2025) (AP) theory, alleles that enhance early-life fitness can be favoured despite imposing costs later in life (Mc Auley, 2025). These mechanisms are not mutually exclusive, and both likely contribute to age related decline. However, while MA and AP help explain how aging may evolve, they do not by themselves provide a sufficiently detailed account of the cellular and physiological processes through which phenotype emerges (Mc Auley, 2025). This limitation is especially important for the present paper. If the aim is to understand why diverse age-related disease arise through a common aging process, and why interventions supposed to delay aging may diverge in efficacy, then evolutionary theory must be connected to more concrete proximate explanations of decline. For this reason, our discussion now turns to more physiologically explicit traditions which likewise situate aging within declining late-life selection, but offer stronger accounts of how age-related decline and pathology are produced.

**FIGURE 2:**
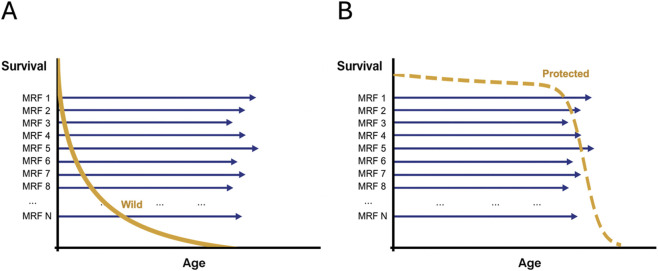
Survival as a function of age under wild and protected conditions, illustrating the role of maintenance and repair functions (MRFs) (Kirkwood, 2005) Maintenance and repair functions (MRFs) refer to cellular and molecular processes–such as DNA repair, proteostasis, and autophagy–that sustain somatic integrity and delay physiological decline. The length of each arrow indicates the functional lifespan of a given MRF–the duration of time for which it can protect the organism from a certain type of damage. The staggered lifespans of different MRFs reflect how individual maintenance systems fail at different times during organismal life. This figure contrasts survival dynamics under two ecological contexts. **(A)** Under wild conditions, survival (sold gold line) declines rapidly with age due to extrinsic hazards such as predation, infection, and starvation. Few individuals live long enough for MRF failure, an intrinsic aging mechanism, to influence mortality. **(B)** Under protected conditions (*e.g.*, laboratory conditions), extrinsic mortality is reduced, and the resultant survival curve (dashed gold line) allows intrinsic aging to become the dominant life-limiting factor. Longevity is then constrained by MRF failure, leading to stochastic molecular damage accumulation, physiological deterioration, and eventual death. Longevity limits are thus set by the capacity for somatic maintenance. Adapted from (Kirkwood, 2005).

Theories become more useful for the present argument when they connect this evolutionary logic to proximate mechanisms of decline. Kirkwood’s Disposable Soma theory provides an evolutionary account in which aging arises explicitly from a failure of somatic maintenance. The theory suggests that because somatic maintenance must only sustain an organism’s physiological condition for as long as survival in the wild is likely, its allocated investment is inherently suboptimal (Kirkwood, 2005). Stochastic molecular damage (*i.e*., somatic mutation (Vijg and Suh, 2013)) can thus accumulate over time, leading to degenerative phenotypes in the post-reproductive period (Kirkwood, 2005). Figure 2 illustrates how fixed levels of investment into maintenance and repair functions (MRFs) (Box 1) ensure survival within an organism’s expected wild lifespan but become life-limiting when extrinsic mortality is reduced. In this vein, large-scale mutagenesis screens have identified many maintenance-associated genes regulating aging (Hasty et al., 2003). However, a major limitation of these approaches is that mutations shortening lifespan or those linked to progeroid syndromes (rare genetic disorders that mimic features of accelerated aging) may not correspond to life-limiting MRFs in wild-type aging.

A key assumption of the Disposable Soma theory is that aging and its pathologies result entirely from suboptimal buffering of stochastic molecular damage (Kirkwood, 2005). The burden of accumulating stochastic molecular damage can account for cell-autonomous aging and clearly drives pathophysiology in certain age-related pathologies, particularly the neurodegenerative conditions (Kumsta et al., 2017; Amanullah et al., 2017). In Parkinson’s disease, the aggregation of amyloid-like α-synuclein in dopaminergic neurons of the substantia nigra pars compacta leads to an accumulation of neurotoxic oligomers, cell death, and loss of inhibitory tone on the basal ganglia (Kouli et al., 2018). Mitochondrial dysfunction also drives dopaminergic cell death in idiopathic and familial Parkinson’s (Kouli et al., 2018). In familial forms of the disease, key implicated genes such as PINK1 and Parkin are components of the mitophagy pathway, and their loss-of-function leads to impaired mitochondrial quality control with resultant cell death (Kouli et al., 2018). Whilst wild-type pathology emerges in the absence of such defined genetic lesions, these cases can be seen as proof-of-principle of the consequences of compromised somatic maintenance.

These damage driven processes, which are readily apparent in the pathophysiology of other neurodegenerative conditions such as ALS (reviewed succinctly here (Rizea et al., 2024)), exemplify the Disposable Soma framework; pathology arises from suboptimally active or overwhelmed somatic-maintenance processes and accumulated cellular damage. Of note, whilst the Disposable Soma framework provides the clearest evolutionary treatment of maintenance-driven aging, late-life maintenance insufficiency need not arise exclusively through trade-offs in resource allocation. The developmental theory of aging can also accommodate maintenance failure through age-dependent failure of somatic maintenance (Box 1). The resulting pathology still reflects accumulated stochastic molecular damage, but in the developmental theory the maintenance deficit arises through suboptimal late-life regulation rather than classical disposable-soma underinvestment.

In contrast, stochastic molecular damage is harder to implicate as the primary driver of pathogenesis in other age-related conditions. This is particularly evident where pathology manifests at a system-wide level, as in cardiovascular aging and disease. An illustrative example is left-ventricular hypertrophy, an independent risk factor for stroke, heart failure, sudden cardiac death, and all-cause mortality (Artham et al., 2009). Left-ventricular hypertrophy begins as an adaptive increase in cardiomyocyte size and protein synthesis in response to chronic pressure or volume overload, mediated by neurohumoral and growth-signalling pathways (Caturano et al., 2022). Only when these programmes remain chronically engaged does the phenotype progress to cell death, fibrosis, mitochondrial dysfunction, impaired protein and mitochondrial quality control, metabolic remodelling and, ultimately, heart failure, arrhythmias and death (Caturano et al., 2022). Thus, in left-ventricular hypertrophy the primary lesion is the sustained activation of otherwise physiological growth and remodelling pathways. Molecular damage emerges as a downstream consequence, illustrating the limits of a purely Disposable-Soma-like account for cardiovascular aging. More broadly, such cases highlight the need for an explanation of why age-related disease processes can be initiated independently of stochastic molecular damage.

The insufficiency of damage-based explanations in accounting for system-level age-related diseases is the impetus for Blagosklonny’s thesis (Gems, 2022). The author aims to provide an ultimate explanation for aging that maintains a central claim: aging and age-related pathology exist along the same continuum, driven by the same underlying mechanisms (Gems, 2022). On this view, lifespan limitation and morbidity are not separate phenomena but reflect different manifestations of a single aging process. Indeed, recent multi-trait polygenic studies of longevity demonstrate that survival is organised not around abstract aging factors, but around genetic liabilities distributed across domains of disease risk and physiological reserve (Jabalameli et al., 2024). A satisfactory theory of aging must therefore explain all the diverse pathologies that accompany aging. Blagosklonny notes that many aging pathologies, such as cancer, atherosclerosis, and diabetes, are in fact diseases of “hyperfunction” (Blagosklonny, 2008). Though focused on TOR (target-of-rapamycin), a conserved master regulator of growth/proliferation (Saxton and Sabatini, 2017), Blagosklonny’s arguments are applicable to the broader endocrine network regulating development and growth, including insulin and insulin-like growth factor signalling (IIS) (Gems, 2022; Lind et al., 2019). These pathways drive pathology through promotion of hyperplasia, dysplasia, hypertrophy, and hypersecretion (Gems, 2022) (Box 1). As pathophysiological mechanisms, these processes are distinct from the stochastic damage accumulation predicted by the Disposable Soma theory. Crucially, as illustrated by left-ventricular hypertrophy, disease initiation can occur in cells whose core functions remain largely intact. In the hyperfunction model, the continued action of growth and developmental programmes, signalling, and effectors beyond when they are required drives aging (Gems, 2022). Further support for the hyperfunction model comes from observations on the correlation between pace-of-life and longevity. Pace-of-life refers to the overall tempo at which an organism develops, grows, and metabolise. This tempo, itself determined by activity of growth and developmental programmes, remains one of the strongest predictors of lifespan and healthspan both between and within species (Yuan et al., 2023). The strong correlation between pace-of-life and longevity therefore strongly implicates the mediators of growth and development in longevity determination.

The evidence-based success of maintenance-insufficiency accounts, such as the Disposable Soma theory, may stem from the fact that many hyperfunction mediators, such as TOR, are themselves negative regulators of MRFs. Inhibition of TOR disinhibits autophagy, an essential MRF, and many studies of life- and healthspan link ablation of the TOR pathway to longevity through autophagy upregulation (Nakamura and Yoshimori, 2018). This conclusion, however, overlooks a key confounder: the simultaneous loss of TOR’s positive effectors. Surprisingly few studies have directly tested whether autophagy is required for the protective effects attributed to TOR suppression. Where this question has been examined, the findings indicate that autophagy is not always required for the protective effects of TOR inhibition. For example, in a myocardial ischaemia–reperfusion injury model in mice, rapamycin confers robust cardioprotection even when autophagy is pharmacologically blocked with 3-methyladenine (Yang et al., 2010). Such autophagy-independent effects are rarely interrogated experimentally, leaving a gap in our understanding of how TOR inhibition mediates protection across tissues and pathologies independently of its canonical effect of autophagy disinhibition. These findings suggest that suppression of TOR’s positive growth- and hyperfunction-associated outputs should seriously be considered as a factor contributing to rapamycin’s effects.

Neither the Disposable Soma theory nor the Hyperfunction theory alone is sufficient to interpret the diversity of age-related phenotypes and pathologies. We argue, however, that the mechanistic emphases foregrounded by these traditions can be productively considered together. Recent work by Le Maître and colleagues helps clarify why such coexistence is formally legitimate. In their broader hierarchical framework, the Disposable Soma theory and developmental theory of aging (including Hyperfunction theory) are treated as compatible evolutionary models that can be situated within a common structure linking genes, physiology, phenotypes, vital rates, and fitness (Lemaître et al., 2024). Our justification for integration here is complementary but independently derived. Rather than seeking broad theoretical integration, we emphasise that aging and age-related pathology lie on the same continuum, and that any upstream explanation must be capable of encompassing the different pathological processes that accompany aging.

**FIGURE 3:**
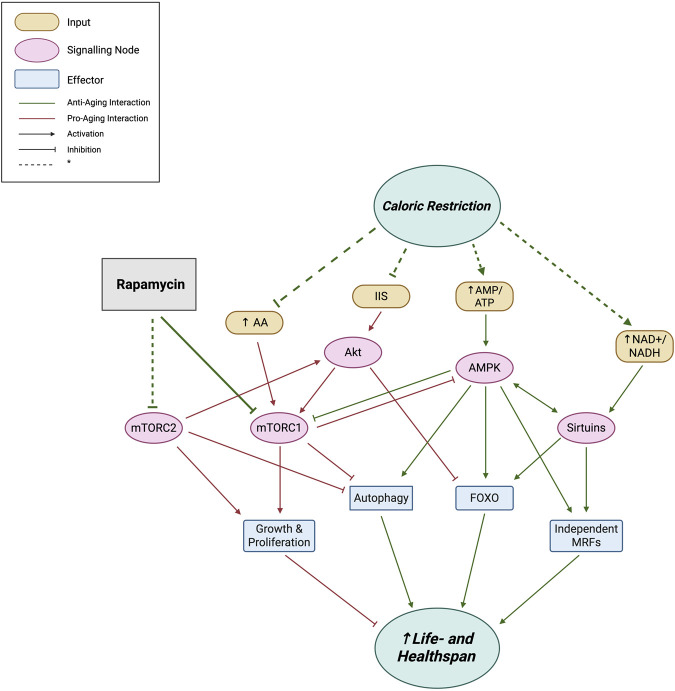
The nutrient-sensing pathway as a mediator of caloric restriction (CR) and rapamycin in aging. CR influences multiple upstream metabolic inputs, including insulin/insulin-like signalling (IIS), amino acid (AA) availability, and AMP/ATP and NAD+/NADH ratios. These signals converge on key nodes such as mTORC1, AMPK, Akt, and sirtuins, which in turn regulate downstream effectors including autophagy, FOXO transcription factors, and other maintenance-and-repair functions (MRFs). CR extends life- and healthspan by simultaneously suppressing pro-growth signals and activating protective effectors. Rapamycin is shown for comparison as a narrower TOR-centred intervention: acutely, it acts primarily as a relatively selective mTORC1 inhibitor, whereas chronic exposure can in some contexts also disrupt mTORC2 signalling. mTORC2 is included to reflect its role in Akt-FOXO regulation and in the interpretation of chronic rapamycin effects, rather than as a uniformly established primary mediator of CR. In Aging Onion terms, hyperfunction refers to pathology arising from persistently elevated growth-, signalling-, or biosynthetic activity, whereas MRF-linked pathology refers to aging processes shaped by suboptimal activation of maintenance and repair programmes. The comparative implication is that, although TOR inhibition may relieve some constraints on MRF activity, CR is better positioned to engage maintenance-promoting mediators in a broader metabolic context, and may therefore address aging processes not fully captured by TOR inhibition alone * *Dotted lines denote effects whose magnitude vary contextually. In the case of CR, nutrient composition of the regimen will differentially modulate the depicted nutrient-sensing inputs. In the case of rapamycin, the interaction with mTORC2 is dependent on chronic exposure and is local tissue or cellular context.* Created in BioRender (Aggour, 2026).

Viewed through this lens, the pathological processes that accompany aging draw explanatory support from both persistent growth signalling, and suboptimal somatic maintenance and repair. Although the latter has been articulated most fully within the Disposable Soma tradition, the developmental account can also accommodate maintenance failure through age-dependent dysregulation in the context of declining selection (so called ‘hypofunction’) (Lemaître et al., 2024). We therefore treat maintenance-insufficiency not as a commitment to a single ultimate explanation, but as a proximate biological axis defined by late-life insufficiency of somatic maintenance. Additionally, Figure 3 shows how some mediators of hyperfunction can reciprocally inhibit maintenance and repair functions. It follows that antagonism between growth and maintenance can be embedded into the evolved mediators of development and growth. These observations suggest that distinct evolutionary explanations may map onto overlapping regulatory architecture, such that persistent growth signalling and insufficient maintenance together could provide a broader upstream framework for interpreting age-related pathology and intervention response.

We conceptualise the integration of hyperfunction and maintenance-insufficiency as an “Aging Onion” (Figure 1) - adapting Gems’ (Gems, 2022) proposal that aging may be understood as a series of layered, life-limiting processes. The Aging Onion is structured at two levels. First, it proposes two broad biological axes of age-promoting process; hyperfunction and maintenance-and-repair insufficiency (Box 1). Second, within each axis sits more specific mechanistic layers. By a layer, we mean a mechanistically distinct and causally relevant vulnerability that can contribute to age-related pathology, can in principle be indexed by measurable variables, and may be differentially engaged or left unresolved by intervention. Layers that fall under hyperfunction would represent variables indicating persistent or excessive programmatic activity, such as sustained anabolic signalling, hypertrophy, hyperplasia, hypersecretion, or chronic remodelling states. Maintenance-insufficiency layers would map onto MRF-related variables, such as impaired mitochondrial quality-control, autophagy, heat-shock response, proteostatic capacity, and DNA damage repair. In the present paper, the Aging Onion is not intended as a fixed quantitative ordering of aging-relevant processes, since the relative contribution of particular layers will vary across tissues, genetic background, and species. Rather, it is a schematic, layered, two-axis framework that organises age-related biology around hyperfunction and maintenance-insufficiency as separable but interacting determinants of age-related pathology. This in turn suggests that interventions aimed at layers contained predominantly within one axis may leave another unresolved, whereas broader strategies may need to engage both hyperfunction and maintenance-insufficiency.

## 3 Why caloric restriction matters: a test case for the Aging Onion

Having outlined the formulation of the “Aging Onion” on conceptual grounds, we explicitly consider how this framework can be probed. Below, we summarise the evolution of the CR response, and why it serves as a natural case study for assessing the interpretive power of the Aging Onion.

Evolution optimises the balance between growth, reproduction, and somatic maintenance, to shape species-specific life histories that determine life- and healthspan (Kirkwood, 2005; Yuan et al., 2023). In many species this balance is, to varying degrees, plastic during the life of an organism. Nutrient scarcity tends to trigger a conserved pro-longevity response across taxa: yeast, roundworms, rodents, and primates show life- and healthspan extension under CR (Speakman and Mitchell, 2011). While the Disposable Soma and Hyperfunction theories offer different mechanistic rationales for this response (Kirkwood, 2005; Gems, 2022), its consistent empirical effects suggest that central aging architecture remains environmentally modulable. We note however that the generality of this response is contested. Deere and colleagues (Deere et al., 2023) raise the important point that the CR literature is heavily concentrated in short-lived model species and often examined under conditions of limited ecological realism. Their work highlights the fact that the CR effect, though seemingly conserved, shows significant heterogeneity, a point which is discussed in more detail below. This capacity for adaptive plasticity nonetheless underpins the interest in CR as a longevity intervention. The nutrient sensing pathway acts as a central hub in this process, and translates environmental signals into coordinated changes in growth, metabolism, and somatic maintenance with aging-relevant effects.

Although caloric restriction consistently extends lifespan across taxa, its effects can be highly context-dependent, varying markedly with species, sex, genetic background, and the degree of dietary restriction imposed. Indeed, a 2012 meta-analysis of 145 studies across 36 species found that CR reduces mortality risk by ∼60% on average, with effect size depending strongly on restriction level and maximal benefit reported at 50% calorie reduction (Nakagawa et al., 2012). Effects of CR were 20% greater in females compared to males and 100% greater in model organisms such as mice and roundworms compared to less-studied species like fish and primates. Therefore, beneath the striking reduction in mortality lies substantial intra- and inter-specific heterogeneity with regards to CR’s effects. CR in *Caenorhabditis elegans* can increase lifespan by upwards of 40% (Smith et al., 2008), and meta-analysis reports a median lifespan increase of 30.4% in rats and 14.6% in mice (Swindell, 2012). Even then, mice show large variation depending on strain and genetic background (Mulvey et al., 2014). The power of CR to regulate aging thus has multiple confounders, the most pronounced being species itself. In *C. elegans*, CR induces a state of extreme metabolic dormancy with drastically enhanced longevity (Braendle and Paaby, 2024) whereas more complex and long-lived organisms – mice, monkeys, humans - appear to exhibit smaller, but still meaningful lifespan extension.

In humans, fasting has long been associated with health benefits anecdotally, but rigorous clinical data remains limited. The CALERIE 2 trial, the first long-term CR study in non-obese humans, showed improvements in biomarkers of health and morbidity risk, but the relatively young cohort limits conclusions on morbidity or lifespan outcomes (Dorling et al., 2021). Turning to primates, two major studies have evaluated chronic CR in Rhesus Monkeys (Mattison et al., 2017), which share 93% sequence identity with the human genome. One study found a significant increase in survival (Colman et al., 2009), while the other did not (Mattison et al., 2012), although methodological differences likely explain the negative result (Colman et al., 2014). Both, however, reported reductions in disease incidence and age-related pathologies (Colman et al., 2009; Mattison et al., 2012; Colman et al., 2014).

The apparent inverse relationship between organismal complexity and the magnitude of CR’s benefits should not be interpreted as a limitation. *Caenorhabditis elegans*’ ability to dramatically extend its lifespan is a product of its life-history strategy (Braendle and Paaby, 2024). Rapid development, early reproduction, and strong selection for plastic responses to environmental stress allow lifespan extension through profound metabolic and developmental suppression. Likewise, the more modest extensions seen in mice and higher organisms likely speak to some evolved developmental restrictions on the upper-limits of physiologically tolerable adaptive lifespan extension in more complex, longer-lived organisms (Kirkwood, 2005). Even so, incremental improvements in human healthspan and morbidity risk would carry substantial clinical and public-health significance. More importantly, CR’s mechanistic underpinnings represent an inroad to the architecture of conserved biological processes that can regulate aging (Speakman and Mitchell, 2011).

CR poses a promising avenue of exploration. This is so because, should CR simultaneously regulate multiple aging processes, it may represent an evolutionarily conserved point of intervention, and a mechanistic benchmark for intervention assessment. Additionally, it acts through a domain which naturally lends itself to Aging Onion style analysis. The nutrient-sensing pathway translates environmental signals into coordinated but distinct changes in growth, metabolism, and maintenance, and therefore allows us to ask whether the layered logic of the Aging Onion is informative. Within the nutrient-sensing literature, targeted pathway inhibition prevails (Lamming et al., 2013). The most prominent example is rapamycin, an mTORC1 inhibitor, which extends lifespan in several model organisms and ameliorates age-associated phenotypes in mammals (Lamming et al., 2013; Johnson et al., 2013). However, if aging is shaped by multiple interacting layers, targeting a single pathway such as TOR may be insufficient to engage the broader range of processes needed for robust geroprotection.

At this point, two related but distinct questions arise. One is whether differences in efficacy between interventions such as CR and rapamycin reflect differences in the breadth with which they engage layered aging-relevant biology. The other is why the same intervention, CR, can produce different effects across strains, sexes, or species. The present paper is concerned primarily with the first question. Interpreting variation in CR responsiveness across biological contexts through the Aging Onion would require independent stratification of hyperfunction- and maintenance-related processes in those contexts and therefore lies beyond the explanatory scope of the framework at this stage.

In the section that follows, we therefore use CR and its leading pharmacological mimetic, rapamycin (Lamming et al., 2013), to evaluate whether the Aging Onion model can map these interventions onto different mechanistic layers of aging and thereby explain their differential performance. We review three lines of evidence to substantiate this discussion: the structure of the nutrient-sensing pathway, outcomes in model systems, and gene expression analysis.

## 4 Comparative evaluation of caloric restriction and rapamycin

### 4.1 The nutrient sensing pathway

The nutrient sensing pathway integrates information about nutrient availability and cellular energetic state to regulate the balance between growth and maintenance (Figure 3). Since both CR and rapamycin act through this pathway, we use it to examine whether their distinct modes of action plausibly correspond to differential engagement of Aging Onion layers and axes. For the purposes of this discussion, the pathway’s key components can be grouped into three levels. First, upstream inputs such as insulin/insulin-like signalling (IIS), amino acid availability, AMP/ATP ratio, and NAD+/NADH ratio convey information about the external and intracellular metabolic environment. Second, these inputs are interpreted by a set of central signalling nodes, principally Akt, mTORC1, mTORC2, AMPK, and the sirtuins. mTORC1 primarily promotes anabolic growth programmes, including protein synthesis, cell growth, and suppression of autophagy, whereas mTORC2 appears to regulate a broader but less well-characterised set of downstream programmes related to metabolism, survival, proliferation, and selected stress-response pathways (Lamming et al., 2012; Sarbassov et al., 2006; Jimenez et al., 2022). Third, these nodes converge on downstream effectors that shape aging-relevant physiology, including growth and proliferation, autophagy, FOXO-dependent stress-resistance programmes, and other maintenance and repair functions. Within this architecture, caloric restriction (CR) acts as a broad systems-level perturbation that alters multiple upstream inputs simultaneously. Conversely, rapamycin is best understood acutely as a relatively selective mTORC1 inhibitor, although chronic administration can in some contexts also disrupt mTORC2 (Lamming et al., 2012; Sarbassov et al., 2006; Schreiber et al., 2015), the relevance of which will be discussed below. The aim of this section is not to catalogue every interaction within the network, but to identify the features most relevant to how CR and rapamycin may differ in their implications for aging.

A key distinction in CR and rapamycin’s effects lies in how IIS and TOR signalling access downstream maintenance pathways. Akt activation simultaneously promotes mTORC1 signalling and suppresses FOXO transcription factors via phosphorylation, thereby dampening stress response pathways (Brunet et al., 1999). IIS inhibition therefore not only downregulates mTORC1 but also relieves FOXO suppression, allowing for an enhanced somatic maintenance response (Greer and Brunet, 2005). In contrast, Akt-independent mTORC1 inhibition, whether through amino acid (AA) deprivation or acute rapamycin treatment, reduces growth signalling but fails to restore FOXO-mediated longevity pathways (Saxton and Sabatini, 2017; Jimenez et al., 2022). In the acute rapamycin context, mTORC1’s failure to engage FOXO pathways is a key mechanistic distinction which could confer CR with a broader maintenance response.

This contrast becomes less absolute under chronic rapamycin treatment, because prolonged exposure can disrupt mTORC2 assembly and signalling *in vitro* and *in vivo* (Lamming et al., 2012; Sarbassov et al., 2006). Since mTORC2 contributes to Akt Ser473 phosphorylation (Sarbassov et al., 2006), its disruption creates a route by which chronic rapamycin may partially extend into FOXO-linked maintenance biology that acute mTORC1-selective inhibition does not reliably engage (Jimenez et al., 2022). Importantly, this extension is context-dependent and not uniform. Rapamycin-mediated mTORC2 inhibition varies across cell lines and tissues, with responsiveness in mice appearing to depend in part on tissue specific FKBP12:FKBP51 expression levels (Schreiber et al., 2015). Chronic rapamycin may therefore engage a broader set of maintenance-relevant outputs than acute rapamycin, but this engagement remains more conditional and less consistently coupled to FOXO disinhibition than the upstream IIS-mediated FOXO relief produced by CR. Moreover, because mTORC2 also supports growth-promoting outputs, including Myc-dependent cell growth in some contexts (Kuo et al., 2015), its involvement complicates interpretation of chronic rapamycin in both maintenance- and hyperfunction-relevant directions. Accordingly, a cleaner separation between hyperfunction- and maintenance-relevant effects may require alternative mTORC1-selective inhibitors with reduced propensity to engage mTORC2 under chronic treatment. Nevertheless, rapamycin remains the focus of the subsequent discussion because it is the mTOR-directed intervention for which the aging literature is currently most developed.

Alongside IIS and TOR signalling, AMPK (AMP-activated protein kinase) and sirtuins mediate energetic stress pathways which play crucial roles in longevity-regulation. Supporting TOR inhibition, AMPK phosphorylates ULK1 (Unc-51-like kinase 1) to initiate autophagy and activates TFEB (Transcription Factor EB), a master regulator of lysosomal biogenesis (Egan et al., 2011). While TOR controls TFEB’s cytosolic retention (Martina et al., 2012), its transcriptional activation is mediated specifically by AMPK-dependent phosphorylation (Settembre et al., 2011). Concordantly, the longevity phenotype of *C. elegans* TOR mutants depends on intact AAK-2 (Zhang et al., 2019), its ortholog of the AMPK α-subunit, underscoring AMPK’s essential role in mediating the benefits of TOR inhibition. The relationship between TOR and AMPK is not solely one of interdependence but of reciprocal inhibition. Recent evidence indicates that mTORC1 can directly suppress AMPK, such that pharmacological mTOR1 inhibition may activate AMPK even in the absence of altered AMP:ATP ratios (Ling et al., 2020). Importantly, this route of activation is not identical to the canonical energetic stress route. The same study found that phenformin, which raises AMP:ATP ratio, acts additively with TOR inhibition to enhance AMPK signalling (Ling et al., 2020). Taken together, these findings suggest that CR and TOR inhibition can engage overlapping but non-identical routes into AMPK regulation.

CR may therefore recruit a broader AMPK- and sirtuin-linked maintenance programme than TOR inhibition alone. In addition to relieving mTORC1-mediated suppression of AMPK, CR is more likely to engage other energetic and redox inputs, namely, altered AMP:ATP and NAD^+^:NADH states, thereby broadening the downstream effector repertoire beyond that achieved by rapamycin. This distinction is relevant because both AMPK and sirtuins upregulate maintenance-relevant outputs such as Peroxisome Proliferator-Activated Receptor Gamma Coactivator 1-alpha (PGC-1a), a central regulator of mitochondrial biogenesis (Rabinovitch et al., 2017). Sirtuins, particularly SIRT1 and SIRT6, play critical roles in DNA repair and maintenance of genomic stability (Imai and Guarente, 2014; Van Meter et al., 2011). SIRT6, for example, activates PARP1 to promote DNA repair in conditions of oxidative stress (Van Meter et al., 2011). The implication is therefore not that AMPK and sirtuins operate independently of IIS/TOR in any absolute sense. Rather, whereas IIS/TOR inhibition may primarily relieve constraints on MRF activity, CR may engage these mediators in a broader metabolic context that enables active upregulation of maintenance programmes that might otherwise remain suboptimally expressed. Through these regulators, CR may extend its effects into aging processes that TOR inhibition alone may address only incompletely, especially where maintenance and repair insufficiency contributes alongside hyperfunction.

### 4.2 Outcomes in mouse models of disease

Blagosklonny emphasised that evaluating anti-aging interventions requires modelling of specific age-related pathologies, rather than lifespan in isolation (Gems, 2022). Moreover, disease-prone mouse models allow a more granular test of intervention mechanisms, by asking whether benefits arise through suppression of hyperfunction, enhanced damage buffering, or both. Both CR and rapamycin improve outcomes in multiple disease-prone mouse models (Unnikrishnan et al., 2020), yet in some contexts their effects diverge (Hursting et al., 1994; Bhattacharya et al., 2012; Christy et al., 2015; Birkisdótti et al., 2021; Livi et al., 2013; Sharp et al., 2003; Dai et al., 2014). Building on a hypothesis that CR and rapamycin’s diverging efficacies can be mapped to differential engagement of Aging Onion layers, we next examine whether this hypothesis is interpretable in the context of disease-prone mouse models.

While organismal aging is a multilayered process, the pathophysiology of individual age-related pathologies can be driven predominantly by one component of the Aging Onion. For example, stochastic damage accumulation appears to be the dominant force driving the neurodegenerative conditions (Amanullah et al., 2017; Powers et al., 2009). Here, we introduce an exploratory prospective classification schematic to categorise disease processes as hyperfunction-driven or maintenance-insufficiency-driven based on the proximate driver of pathology in the instantiated untreated model (Table 1). This classification is intended as a structured interpretive tool rather than a definitive taxonomy and is assigned independently of intervention outcomes. Of note, we do not intend to provide a definitive ontology of the wild-type correlates of these disease models. The aim is to classify the dominant pathological process foregrounded by the specific model conditions under study. For example, in neoplastic models, distal mutational origins may implicate genome-maintenance failure broadly, but classification here is based on the proximate driver of pathology in the instantiated model. Using this classification schematic, we then looked to see whether the effects of CR and rapamycin vary systematically with the type of pathological mechanism proposed to dominate each model. Any consistent pattern emerging from this analysis would not define the classification, but could corroborate its interpretive usefulness and, in turn, the Aging Onion framework more broadly.

**TABLE 1:** Prospective criteria for classifying disease models as driven by either hyperfunction or maintenance-insufficiency. A schematic to classify disease models through the axes of the Aging Onion. Here, hyperfunction refers to pathology driven primarily by excessive or insufficiently restrained execution of growth/developmental programmes, whereas insufficient maintenance and repair refers to pathology driven primarily by failure of processes that normally preserve cellular or tissue integrity.

Criterion	Question	Characteristics of hyperfunction	Characteristics of maintenance insufficiency
Initiating abnormality	What is the first proximate causal abnormality instantiated in the untreated model?	Constitutive, excessive, unrestrained, and broadly maladaptive activation of a physiological programme (for example, growth, secretory, inflammatory, or remodelling signalling)	Loss or impairment of a function that normally limits damage accumulation or preserves cellular integrity (for example, proteostasis, genome maintenance, mitochondrial quality control, detoxification, general damage surveillance and clearance)
Earliest causal pathology	What is the first pathological sate that follows from that abnormality?	Pathological overactivity of tissue or cells demonstrated by hyperplasia, hypertrophy, hypersecretion, persistent activation, or maladaptive remodelling	Early damage accumulation and associated toxicity, instability, DNA damage, organellar dysfunction, cellular loss. These changes are typically cell-autonomous
Direction of causal flow	In the main pathogenic sequence, does pathological overactivity generate damage, or does primary damage generate later dysfunction?	Excess programme activity is upstream, and tissue stress, injury, or damage appears mainly as a downstream consequence	Damage burden or failed containment is upstream, and later dysfunction, compensatory pathway activation, or remodelling appears mainly as a downstream consequence
Requirements for persistence	In the established untreated pathology, what must continue for the phenotype to persist?	Continued programme activation or continued loss of restraint over that programme	Continued damage burden or continued failure to contain, clear, or repair that burden

Table 2 presents five disease models in which both CR and rapamycin have been tested under comparable conditions, listing outcomes alongside the putative dominant pathological driver. Supplementary Table S1 shows the worked logic used to assign categories using the schematic in Table 1.

**TABLE 2:** Differential effects of caloric restriction (CR) and Rapamycin in disease-prone mouse models. Five mouse models of age-related pathology are shown in which both CR- and rapamycin-treated cohorts were evaluated under comparable conditions. For each model, the dominant pathological mechanism was assigned prospectively using the schematic in Table 1, based on the proximate driver of pathology in the instantiated untreated model. CR and rapamycin outcomes are summarised as reported in the cited studies. The table is intended as a structured comparison of intervention patterns across models classified through hyperfunction or insufficient maintenance and repair labels.

Disease model	Dominant pathology mechanism	CR effect	Rapamycin effect	Reference
Amyotrophic Lateral Sclerosis (SOD1^H46R/H48Q^)	*Maintenance-insufficiency*	*- Delayed disease onset* *- extended lifespan by ∼14%, prolonged motor function*	*- No impact on disease onset, duration, or survival*	Bhattacharya et al. (2012)
Cancer (Rb1^+/−^)	*Hyperfunction*	*- No significant effect on lifespan or tumour growth*	*- Substantial lifespan extension* *- Suppression of pituitary and thyroid tumours* *- Reduced metastasis*	Livi et al. (2013), Sharp et al. (2003)
Cancer (p53^−/−^)	*Maintenance-insufficiency*	*- Delays tumour onset, increases median survival by ∼56%*	*- No lifespan benefit at standard dosing; dependent on intact p53 for tumour suppression*	Hursting et al. (1994), Christy et al. (2015)
Cardiac Aging (26 months)	*Hyperfunction*	*- Strongly decreased left ventricular hypertrophy to youthful levels* *- Reversed myocardial performance to youthful levels* *- Restored diastolic function to youthful levels*	*- Moderately decreased left ventricular hypertrophy* *- Moderately restored myocardial performance* *- Restored diastolic function to youthful levels*	Dai et al. (2014)
Progeroid DNA repair deficiency (Ercc1^Δ/-^)	*Maintenance-insufficiency*	*- Triples lifespan* *- improves neuromuscular function, delays onset of age-related symptoms* *- reduces oxidative DNA damage, transcriptional stress, inflammatory gene expression*	*- No extension of lifespan* *- No improvement in function or reduction of pathology* *- No impact on DNA damage burden* *- No effects despite effective mTORC1 inhibition*	Birkisdótti et al. (2021)

A provisional pattern emerges from the prospectively classified models. In models where pathology is driven primarily by maintenance and repair deficits, such as in *p53* ^*−/−*^ and SOD1^H46R/H48Q^ mice, CR extends lifespan while rapamycin does not (Hursting et al., 1994; Bhattacharya et al., 2012; Christy et al., 2015). Conversely, in the hyperfunction-foregrounding *Rb* ^*+*^ */* ^*−*^ neuroendocrine tumorigenesis model, rapamycin outperforms CR (Livi et al., 2013; Sharp et al., 2003). Taken at face value, this pattern is consistent with the possibility that CR more effectively engages maintenance and repair processes in damage-dominant contexts, whereas rapamycin performs better where the dominant pathology is persistent proliferative or growth-program overactivity and there are no direct lesions to maintenance and repair mechanisms. We note that rapamycin’s known immunomodulatory effects could be viewed as a potential confounder in its observed tumour suppression here. Rapamycin’s canonical immune effects are profoundly suppressive, particularly through inhibition of lymphocyte activation and maturation, which falls cleanly into hyperfunction suppression. However, growing evidence for context-dependent immunostimulatory effects complicates precise mechanistic attribution.

More broadly, a model of cardiac aging, shows that the emerging pattern cannot be read too simply. In geriatric mice with age-associated structural and functional cardiac degeneration, we classified the dominant pathological mechanism as hyperfunction on the basis of causal flow from maladaptive growth-programme overactivity (Supplementary Table S1). This was despite an additional strong presence of damage-associated features in the disease process (Dai et al., 2014). In this study, CR outperformed rapamycin on all but one metric (Dai et al., 2014) (Table 2). A few interpretations here are plausible. First, CR may in this context suppress hyperfunction and associated maintenance/repair failure more effectively than rapamycin alone. Second, assigning a single dominant label to a disease process may obscure mechanistic shifts in the disease course over time, such that the processes most important for disease maintenance are not identical to those that first generated the phenotype. Third, the proposed hyperfunction-versus-maintenance distinction may not be the dominant determinant of intervention response. This diversity of plausible interpretations exposes an important limitation of the current framework. Unless prospective assignment is both well supported and sensitive to shifts in dominant mechanism over the disease course, intervention outcomes cannot be confidently interpreted in relation to the proposed pathological category.

With this limitation in mind, the Aging Onion may be better at describing upstream disease-generating logic than therapeutic point-of-care leverage points. We developed the Aging Onion as a unified, intervention facing framework which looks to capture core features of aging regulation. Placing the framework upstream of more granular descriptors of aging (dys)regulation such as the hallmarks of aging may however carry a translational cost. In practice, the clinical application of aging biology is likely to emerge first in the management of age-related diseases rather than in primary prevention. This reflects aging’s position outside of formal disease classification and the evidentiary and regulatory burdens attached to interventions that seek to modify aging itself (Muscedere et al., 2025). In this setting, assigning broad hyperfunction or maintenance-insufficiency labels to complex pathologies may not yet be sufficiently acute to guide treatment. This is especially the case where dominant vulnerabilities may shift over time or differ across disease components. Distinguishing hyperfunction-dominance from maintenance-insufficiency in individual pathologies likely requires more hypothesis-driven research, ideally with interventional feedback from known suppressors of growth or activators of maintenance and repair applied at different disease stages. In the cancer context, synthetic-lethal logic may sometimes offer such leverage, but elsewhere this is difficult to establish. More downstream and less unified frameworks, for example, at the level of specific aging hallmarks or persistence requirements, may be of greater therapeutic traction once disease is established. By contrast, the Aging Onion framework may be better placed in the prevention space, where its value lies in its attempt to clarify which upstream biological axes are most plausibly being engaged by broader anti-aging interventions.

Even with this limitation acknowledged, the Aging Onion framework remains useful only if its interpretation of intervention outcomes is falsifiable. Because CR is hypothesised to act across both hyperfunction and maintenance/repair layers, CR superiority is not by itself strongly diagnostic. The more important differentiating pattern is whether CR shows increasing relative advantage as pathology becomes more damage/MRF-failure-dominant. Rapamycin superiority, or parity with CR, remains informative: if such cases localise to confidently assigned hyperfunction-foregrounding models, that is consistent with the proposed role of selective hyperfunction suppression. However, rapamycin outcompeting CR in confidently assigned maintenance-insufficiency-dominant models would weigh directly against the proposed mechanistic split and undermine the interpretive power of the Aging Onion.

These twin requirements – clearer pathological assignment and cleaner outcome interpretation – point toward the need for stronger mechanistic pole models, especially if the Aging Onion is to remain informative as an account of upstream aging drivers. If hyperfunction is to add any intervention-facing value to the framework, some pathologies must be identifiable in which it is clearly foregrounded on prospective mechanistic grounds. Such cases are needed not because rapamycin must outperform CR in them, but because they provide a cleaner setting in which rapamycin’s comparative performance can be interpreted mechanistically rather than being obscured by mixed pathology. For this purpose, we suggest the use of progeroid models as a mechanistic probe. At one extreme, progeroid DNA-repair-deficient models such as Ercc1-/Δ provide a relatively clean example of damage/maintenance-insufficiency-dominant pathology (Supplementary Table S1), in which accelerated lesion accumulation and genome-maintenance failure are clearly foregrounded (Wong et al., 2020). In this model, CR has been shown to significantly outperform rapamycin (Birkisdótti et al., 2021). At the other end, endocrine-overdrive models such as bovine growth hormone (bGH) transgenic mice (Supplementary Table S1) may offer a clearer hyperfunction-foregrounding case, in which persistent activation of physiological growth programmes drives systemic overgrowth, metabolic dysregulation, and downstream organ pathology (Ding et al., 2013; Bartke et al., 2002). We believe a comparison of CR and rapamycin in such a model, where hyperfunction is dramatically foregrounded, could help clarify the relevance of the hyperfunction axis to intervention outcomes. Progeroid models are by no means naturalistic replicas of wild-type aging, but they may nevertheless provide more interpretable test systems for asking whether CR and rapamycin diverge systematically across more clearly separated mechanistic settings. In that sense, their value is not that they prove the Aging Onion directly, but that they provide cleaner conditions in which its distinctive claims can either be supported or challenged.

In summary, an Aging Onion informed view that CR outperforms rapamycin due to broader engagement of both hyperfunction and maintenance axes is defensible so long as CR shows increasing relative advantage as pathology becomes more maintenance-insufficiency-dominant. This trend is reflected in Table 2 but is by no means definitive because the available disease-model comparisons are limited. Stronger support will require additional experiments in models that more clearly foreground one axis or the other. Within that context, rapamycin superiority in a clearly hyperfunction-foregrounding model is not necessary for the framework to remain tenable, but it would materially strengthen the claim that hyperfunction can in some settings become sufficiently foregrounded to shape comparative intervention efficacy.

### 4.3 Gene expression analysis

Across this paper, we have argued that aging may be more usefully understood as involving both hyperfunctional processes and insufficient maintenance and repair, and that this distinction may help explain why CR often exerts broader anti-aging effects than rapamycin. The preceding sections suggest that this interpretation is consistent with both the organisation of nutrient-sensing pathways and differential intervention outcomes in model systems. However, the value of the Aging Onion framework depends not only on its conceptual coherence, but also on whether its proposed mechanistic distinctions can be related to quantitative biological outputs. Gene expression analysis offers one such exploratory lens, through which patterns of intervention response may be examined for consistency with the framework’s predictions.

A direct way to interrogate the Aging Onion hypothesis is to ask whether CR and rapamycin produce distinct transcriptional programmes consistent with differential engagement of hyperfunction and MRF-related biology. For instance, to evaluate the biological programs engaged by CR vs. rapamycin, Fok and colleagues (Fok et al., 2014) conducted comprehensive transcriptomic analysis of liver tissue from male C57BL/6 mice treated with CR, rapamycin, or both for 6 months. Among 25,600 transcripts analysed, ∼2,500 were significantly altered by either intervention but only 20% of these overlapped between groups. Notably, CR had a greater impact on upregulated genes while rapamycin more strongly affected downregulated genes. This distinction may reflect their underlying mechanisms: CR may activate suboptimal MRFs whereas rapamycin more narrowly suppresses overactive hyperfunctional pathways. The authors did not test for alignment into hyperfunction or MRF-like categories. However, upon qualitative inspection of the top 15 IPA-enriched terms, we found that rapamycin predominantly affects canonical growth and biosynthesis pathways, consistent with hyperfunction suppression. Additionally, several CR-associated pathways (protein ubiquitination, mitochondrial dysfunction, epithelial adherens junction signalling, remodelling of epithelial adherens junctions, and FXR/RXR activation) may plausibly constitute MRF upregulation. However, without a formal, conceptually grounded classification of MRF-related pathways as pertains to the axes of the Aging Onion, it would be premature to interpret these as definitive MRF activation. Rather, the data serves to highlight the need for hypothesis-driven annotation frameworks capable of testing these axes more rigorously.

Interpretation of transcriptomic data in other head-to-head studies of CR and rapamycin is similarly difficult. Zhang and colleagues (Zhang et al., 2025) demonstrated in yeast that CR and rapamycin affect largely distinct gene sets in both mitotic and postmitotic phases. However, the published gene-ontology term summary is restricted to the most enriched pathways rather than the full functional spectrum. In these top hits, a bias towards hyperfunction suppression or MRF activation in either treatment is not readily evident. Likewise, in Ham and colleagues’ (Ham et al., 2022) study of skeletal muscle in geriatric mice treated with either CR or rapamycin, top pathways are not clearly interpretable within our framework and mainly show divergences in metabolic reprogramming. This is not to say that Aging Onion’s categorisation does not have explanatory power, but rather that, in this tissue context and given the selective representation of pathways in figure panels, the data are not readily interpretable. The hypothesis of the Aging Onion is that across organisms and tissue types, intervention success could be mechanistically and quantitatively interpretable through differential engagement of hyperfunction and MRF programs. Validating this hypothesis would require omic datasets to be screened using these axes explicitly, with careful, biologically justified *a priori* classification of pathways into either category.

Mechanistic clarity can improve when model systems are designed to probe a specific limiting process. A compelling insight arises from Ham’s (Ham et al., 2022) use of a constitutively active mTORC1 mouse model in which proteostasis is compromised, as evidenced by p62 accumulation. In this setting, CR alleviates sarcopenia via upregulation of Xbp1 (a UPR transcription factor), Keap1 (a regulator of the NRF2 oxidative stress response), and the resulting reduction in p62 burden. Because the model renders maintenance failure overt, the experimental design becomes a functional screen for interventions that restore somatic resilience. The success of CR at alleviating sarcopenia under these conditions, despite persistent mTORC1 signalling, demonstrates its capacity to engage MRFs independently of TOR effectors (Ham et al., 2022). Thus, when maintenance failure is experimentally foregrounded, CR’s engagement of MRFs becomes both detectable and relevant. This observation underscores the broader methodological point: full transcriptomic datasets from CR- or rapamycin-treated models may harbour testable signals of mechanistic divergence but require hypothesis-driven pathway categorisation to be meaningfully interpreted.

## 5 Future directions: Empirical agendas for testing and applying the Aging Onion

The preceding sections suggest that the Aging Onion may help organise why interventions acting through nutrient-sensing biology differ in breadth and efficacy. Here, we outline two future research agendas. The first considers whether, and if so how, transcriptomic data could be used to interrogate how candidate interventions engage hyperfunction- and maintenance-related domains. The second asks whether the framework can be justifiably generalised beyond nutrient-sensing biology, including onto the broader genetic determination of lifespan and healthspan.

### 5.1 A transcriptomic research agenda for intervention stratification and evaluation

Above, we highlighted the potential for the Aging Onion model to be tested against transcriptomic datasets to determine whether it can explain or predict intervention performance. To assess whether such a methodology could be of use in anti-aging intervention assessment and, in time, discovery, it is helpful to first take a brief tour of the current landscape.

The ideal geroprotector should not only increase lifespan but also attenuate biomarkers of aging and improve healthspan (Moskalev et al., 2017). Several pharmacological candidates have been advanced on the grounds that they reproduce elements of CR’s input into the nutrient-sensing pathway. These include rapamycin, metformin, NAD^+^ boosters, and more recently the blockbuster GLP-1 receptor agonists (GLP-1RAs) (Guarente et al., 2024). Against this backdrop, the National Institute on Aging’s Interventions Testing Program provides an important benchmark (Strong et al., 2024). Screening a broad panel of candidate geroprotectors in genetically heterogeneous mice, the Interventions Testing Program has consistently found CR to outperform other interventions, whether alone or in combination. Notably, newer candidates such as the GLP-1RAs have not yet been included in Interventions Testing Program analyses, yet their widespread clinical use and emerging evidence of morbidity reduction (Perkovic et al., 2024; Kosiborod et al., 2023; Lincoff et al., 2023; Marso et al., 2016) make them important to contextualise. Because appetite suppression is a major component of their action (Zheng et al., 2024), GLP-1RAs are relevant to comparison with CR-like interventions. However, recent work in preprint shows that low-dose exenatide can reverse omic aging signatures in aged mice through central hypothalamic signalling, without any changes in food intake or body weight (Huang et al., 2024). It is precisely these cases which would benefit from more structured analysis in future - geroprotective effects are apparent but the breadth and basis of engagement with aging biology remain unsolved. In particular, it is unclear whether hypothalamic GLP-1R agonism, in the absence of reduced energy intake, can recapitulate the breadth of nutrient-sensing-driven geroprotection engaged by true CR.

These uncertainties reinforce the need for discovery workflows that can capture the full breadth of CR’s effects while also situating emerging candidates such as GLP-1RAs within a mechanistically grounded framework. This tension is especially visible in transcriptomic drug discovery, where candidate compounds are often prioritised by their ability to reverse age-associated expression changes without explicitly imposing a mechanistic framework on those changes. For instance, Donertas and colleagues (Dönertaş et al., 2018) compiled gene expression profiles from multiple datasets of aged human brain tissue to identify genes whose expression changed with age. They then queried CMap to identify drugs that induce opposing transcriptional effects. This approach, which focuses on global reversal of age-associated gene expression signatures, identified 24 candidate compounds, including several known pro-longevity agents. However, the authors note that this strategy cannot distinguish between adaptive vs. maladaptive age-related expression changes. A refinement came with the ANDRU pipeline (Yang et al., 2020) which began by constructing co-expression networks from transcriptomes of young and old human adipose tissue. The workflow then identified age-perturbed subnetworks that converge with age-related disease signatures. Drug perturbation databases were subsequently queried to find compounds that reverse these specific subnetwork level changes. The workflow thus narrows the scope from global differentially expressed genes to those which are likely relevant to the mechanisms of age-related disease in the tissue in question. While these methods have produced plausible leads, both rely on (high-dimensional) statistical correlation between transcriptomic states without explicit integration of *what kind* of biological processes are being reversed or engaged.

Some efforts have used the transcriptional signature of CR as a benchmark to screen for drugs with similar profiles - leveraging CR’s conserved anti-aging effects. This approach avoids some pitfalls of simple signature reversal by anchoring candidate selection to an empirically validated pro-longevity intervention rather than to general aging-associated change. However, similarity to CR at the level of the whole-transcriptome does not reveal *which aspects* of CR biology are being mimicked. Calvert and colleagues (Calvert et al., 2016) provide an instructive example of this limitation. The group used a curated transcriptional signature derived from CR treated rat and monkey cells to query for drugs with similar profiles. Their strongest match was rapamycin, a drug that, as discussed before, affects largely distinct gene sets (Fok et al., 2014; Zhang et al., 2025; Ham et al., 2022), engages divergent pathways (Fok et al., 2014; Zhang et al., 2025; Ham et al., 2022), and which ultimately fails to recapitulate CR’s effects in models (Unnikrishnan et al., 2020). Without dissecting which aspects of CR biology are being mimicked, this approach allows compounds with very different mechanisms, and inferior performance, to score highly if their net transcriptomic effects align with CR on aggregate.

The potential value of the Aging Onion in this setting would not be to generate another global similarity metric, but to ask whether intervention responses can be resolved into more defined biological axes, and whether those axes help explain differences in comparative performance. In this context, the relevant distinction is between (i) continued or excessive activity of developmental and growth programmes (hyperfunction), and (ii) inadequate somatic maintenance and repair. This could move comparison beyond simple reversal of age-associated signatures or aggregate similarity to CR, toward a more mechanistically structured account of intervention breadth.

An important proof-of-principle case would be the CR-versus-rapamycin comparison discussed throughout this paper, ideally in matched tissues and timepoints alongside untreated young and aged controls. This would allow three questions to be asked:

1. Can age-related transcriptomic perturbations in a given tissue be organised in terms of hyperfunction-associated versus maintenance-related biology?
2. Do CR and rapamycin modify those same axes in different ways?
3. Does stratification of CR and rapamycin according to the direction and relative magnitude of those differences help explain their observed divergence *in vivo* efficacy?

Any such effort would need to be framed primarily at the level of transcriptional programmes or grouped functional domains rather than individual genes, given the likely pleiotropy and context specificity involved. A defensible starting point would therefore be to define, in advance, programme-level signatures that are intended to capture hyperfunction-associated and maintenance-related biology (Box 1), then ask how aging shifts the balance between them in a given tissue context. Here, age-associated expression is most useful as a reference against which to compare intervention-induced perturbation, rather than as a fully interpretable domain in its own right. This is especially important for maintenance insufficiency, since, depending on the ultimate explanation invoked, late-life maintenance can be suboptimal even without age-related loss of expression (Box 1). The more relevant question is therefore whether interventions move the Aging Onion’s axes in a favourable direction, by reducing hyperfunction-associated activity, engaging maintenance-related processes, or both.

In a proof-of-principle setting, the value of this approach would depend on whether interventions can be stratified according to the direction and magnitude of their engagement with the Aging Onion axes, relative to the aged-control. This stratification could then be compared against independently observed, non-transcriptional differences in performance relevant to the tissue or model under study, such as disease modification or reduction of local pathology. If the axes of the Aging Onion can stratify CR and rapamycin signatures in a way that matches their observed differences in efficacy, the framework could thereafter be extended to other nutrient sensing interventions before any broader application is attempted. Equally, failure of this framework to distinguish CR from rapamycin in a way that matches observed non-transcriptional differences in efficacy, or the recurrent emergence of important transcriptomic dimensions not captured by the hyperfunction/MRF distinction, would place clear limits on its usefulness in this setting.

### 5.2 Testing the broader generalisability of the Aging Onion

Until this point, the Aging Onion has been examined primarily within nutrient-sensing biology. This restriction has been deliberate. The framework was not introduced as a universal taxonomy of aging, but as a unified pathology- and intervention-facing model, situated within broader hierarchy of aging causation, and intended to organise why different age-related vulnerabilities arise and why interventions differ in breadth and efficacy. The nutrient-sensing domain is where the comparative and layered logic of the framework is most easily interpretable – and where CR versus rapamycin contrasts could offer initial proof-of-principle. Above we proposed applying the Aging Onion to transcriptomic analysis of CR versus rapamycin and, thereafter, other nutrient-sensing interventions. Beyond this setting, however, broader generalisation should not be assumed but directly tested. One external arena in which this is possible is the genetic architecture of lifespan and healthspan.

Large-scale population-based human genetic studies already suggest that lifespan and longevity are highly polygenic, and that the variants influencing them are pleiotropic and distributed across multiple health-related domains (Timmers et al., 2019; Deelen et al., 2019). This diffuse genetic architecture is a ripe testing ground for any framework that claims to organise aging more coherently. The question for the Aging Onion is whether it retains explanatory value in this setting and if genetic architecture of longevity can be captured by the two axes that the framework currently describes. Jabalameli and colleagues (Jabalameli et al., 2024) provide a direct bridge to the kind of analysis the Aging Onion is suited for. Rather than treating longevity as a phenotype explained by many small direct genetic hits, they model it as the combined outcome of many genetically correlated liabilities across aging-related traits and biomarkers. Their integrated longevity genetic score was built from the combined polygenic risk for dozens of traits, including cardiovascular, respiratory, reproductive, cognitive, metabolic, immunological and skeletal health domains, together with interaction terms between them (Jabalameli et al., 2024). In this sense, the study goes beyond polygenicity to operationalise longevity as a composite outcome of multiple interacting trait-liabilities. The success of their analysis is encouraging for the Aging Onion framework, which proposes similar structural claims, namely, that aging reflects liability distributed across two major biological axes. Furthermore, one could ask whether the trait dimensions analysed in Jabalameli’s work can be organised in terms of hyperfunction-like and maintenance-like liabilities. Traits that resist organisation into these categories would be equally informative, because they would indicate dimensions of aging biology that the Aging Onion does not currently capture.

There are already indications in Jabalameli’s analysis that an application of the Aging Onion to the genomic data would be fruitful. In their proteomic follow-up, the authors identified plasma proteins associated with the integrated longevity genetic score and used DrugBank to search for interacting proteins with repurposing potential (Jabalameli et al., 2024). This analysis highlighted Fostamatinib, a spleen tyrosine kinase (SYK) inhibitor which sits upstream of PI3K-Akt signalling. Since PI3K-Akt is one of the central nodes of the nutrient-sensing pathway and a major regulator of the growth/maintenance biology, its emergence here is more than incidental. It suggests that the diffuse genetic architecture of longevity may converge on higher-order regulatory hubs that sit upstream of trait-level liabilities. In that respect, the result is at least preliminarily supportive of the Aging Onion’s claim that aging can be organised around the intervention-relevant biological axes of growth/development and somatic maintenance.

The relevant next steps would therefore be to ask whether the major trait-liability domains underlying longevity can be meaningfully organised in terms of hyperfunction-like and maintenance-like biology using a schematic similar to that in Table 1. Where categorisation remains unclear, second-order analysis of the proteomic correlates of the integrated longevity genetic score could provide additional guidance as to whether the underlying biology leans more toward growth-associated or maintenance-related processes. Where ambiguity recurs, however, it should be seriously considered as evidence that additional biological axes may be relevant and that the Aging Onion requires expansion for the purpose of generalisation.

## 6 Conclusion

Aging research has generated increasingly detailed accounts of the molecular and physiological changes that accompany decline, yet a coherent framework linking these proximate processes to evolutionary theory and intervention logic remains underdeveloped (Kennedy et al., 2014). In this paper, we have proposed the ‘Aging Onion’ as a layered, two-axis framework organised around hyperfunction and maintenance-insufficiency (Box 1). Rather than treating the Disposable Soma and Hyperfunction traditions as mutually exclusive, we argue that their mechanistic emphases represent distinct but compatible axes of age-promoting process. These axes are insufficient somatic maintenance and repair, and persistent activity of growth and developmental programmes beyond their adaptive window. The value of the Aging Onion is to provide a framework for how different age-related pathologies and intervention responses may reflect multiple interacting mechanistic layers organised along two biological axes. On this view, targeting one limiting process may leave another unresolved, and the efficacy of an intervention may depend in part on both the number and type of relevant layers it engages. We used nutrient sensing as an initial benchmark domain through which to explore the Aging Onion’s logic, specifically through a comparison of CR and rapamycin. We argue that their differences across pathway engagement, model systems, and transcriptomic evidence, are consistent with the possibility that CR’s greater efficacy reflects broader engagement of both hyperfunction and maintenance-insufficiency layers than rapamycin.

We suggest that the framework may be useful for organising mechanistic differences between interventions and for interpreting why their efficacies diverge. However, due to limitations in the current evidence-base we propose future work to clarify important points. One direction is to determine whether the framework proposed here can be operationalised in intervention assessment, including through more systematic use of transcriptomics. Another is to determine whether the layers and axes of the Aging Onion generalise beyond nutrient sensing to other domains of aging biology, including the polygenic and population-genetic architecture of lifespan and longevity. The central question is whether the Aging Onion can reliably capture the biological complexity of aging while remaining useful for intervention-oriented geroscience.

BOX 1 **Biological axes of the aging onion.**

For the purposes of the Aging Onion framework, we define here two broad biological axes used throughout the paper and note the evolutionary traditions that most strongly foreground them. We focus on these axes as proximate, pathology- and intervention-relevant categories of processes that promote aging.

**PERSISTENT GROWTH AND DEVELOPMENTAL SIGNALLING–HYPERFUNCTION:**

- **Definition:** persistent activity of growth and developmental programmes beyond their adaptive window promotes aging and its associated degeneration and pathology.
- **Foregrounded by:** Hyperfunction Theory, a derivative of the developmental theory of aging. Hyperfunction reflects evolutionarily unopposed programmatic activity in the post-reproductive period – aging as an unintended pathological continuation of development.
- **Mechanistic Mediators:** Molecular processes driving hyperplasia, hypertrophy, metabolic imbalance, and chronic secretory phenotypes. These states arise from unopposed signalling through growth pathways (*e.g.,* TOR, IGF, PI3K/AKT) and often suppress somatic maintenance processes.

SUBOPTIMAL SOMATIC MAINTENANCE – MAINTENANCE AND REPAIR INSUFFICIENCY:

- **Definition:** Insufficient effective maintenance-and-repair capacity in later life. This may reflect chronically inadequate baseline activity, age-dependent decline in function, or impaired inducible deployment of otherwise intact maintenance and repair functions (MRFs). When MRF activity or deployment is suboptimal, stochastic molecular damage accumulates and cellular integrity progressively deteriorates, contributing to aging and its pathologies in later life. In wild-type aging, this suboptimality does not reflect genetic compromise of core MRF components, but the insufficient activity of otherwise intact maintenance systems over time.
- **Foregrounded by:** most explicitly the Disposable Soma Tradition, in which organisms allocate finite resources to maximise reproductive success rather than indefinitely maintaining the soma. Investment in MRFs is calibrated to support a health soma through the organism’s expected reproductive lifespan, but not thereafter. The developmental theory of aging can also generate late-life maintenance failure through “hypofunction”, where declining selection allows protective functions to remain below their age specific optimum due to age-dependent changes in expression, activation, or inducibility.
- **Mechanistic Mediators:** (suboptimal activity of) MRFs – includes DNA repair, proteostasis, autophagy, organelle repair, detoxification, and antioxidant defence

## Data Availability

The original contributions presented in the study are included in the article/Supplementary Material, further inquiries can be directed to the corresponding authors.
