# Supplementary material for: Integrating evolutionary theory into a framework for the mechanistic evaluation of candidate anti-aging interventions
Source: Front Aging. 2026 Jun 15;7:1800288. doi: 10.3389/fragi.2026.1800288 (PMC13311018; doi:10.3389/fragi.2026.1800288)
Supplement: Supplementary file 1 [file Table1.docx]

| **Model** | **Initiating abnormality** | **Earliest causal pathology** | **Direction of causal flow** | **Persistence Requirement** | **Assignment** | **Caveat** |
| --- | --- | --- | --- | --- | --- | --- |
| **Amyotrophic Lateral Sclerosis (SOD1H46R/H48Q)^1,2^** | Mutant SOD1 maturation/proteostasis defect: H46R/H48Q disrupts normal metal binding and post-translational maturation, producing destabilized off-pathway folding intermediates | Misfolded/aggregation-prone toxic SOD1 species, with fibrillar SOD1-containing aggregates and toxic immature mutant protein preceding degeneration | Damage/proteostasis failure comes first: toxic mutant-protein instability and failed maturation are upstream; motor neuron dysfunction/degeneration is downstream rather than driven by primary programme-like overactivity | Continued burden of toxic mutant SOD1 species and associated proteostatic/mitochondrial stress is required to sustain pathology; this is inferred from the model biology rather than directly isolated experimentally | **Maintenance-insufficiency-dominant** | Best classified at the level of mutant-SOD1 proteotoxic pathology within this instantiated model, not ALS as a whole. “MRF-insufficiency” here mainly refers to proteostasis/maturation failure rather than a clean canonical DNA-damage model; “stochastic damage” is therefore too broad unless qualified. DR benefit and rapamycin non-response may corroborate this interpretation, but do not define it. |
| **Cancer (Rb1+/-)^3–6^** | Loss of heterozygosity (LOH) of the remaining wild-type Rb1 allele, causing RB deficiency in susceptible neuroendocrine cells | Early atypical proliferation (EAP) in pituitary/neuroendocrine tissues, reflecting loss of proliferative restraint and preceding overt pituitary and thyroid tumors | Proliferative dysregulation comes first: RB-deficient atypical proliferates arise early and progress to overt tumors; later genomic instability and stress-related malignant features are secondary consequences or acquired liabilities rather than the primary pathological driver in the model as assayed | Continued RB-deficient proliferative deregulation is central to ongoing tumor growth, but persistence of established tumors likely also depends on secondary stress-adaptation and survival pathways created by RB loss. Thus, maintenance is not reducible to proliferation alone: RB re-expression can induce sustained cell-cycle arrest and suppress ongoing proliferation, yet residual lesion burden persists, consistent with additional downstream dependencies in the established tumour state | **Hyperfunction-dominant** | The proximate initiating abnormality is a genetic lesion, but the model is classified by the pathological process that lesion instantiates, namely loss of proliferative restraint and persistent mitogenic programme execution. Thus, LOH does not by itself make the model damage-dominant. Best classified at the level of the instantiated tumor model, not wild-type aging generally. |
| **Cancer (p53-/-)^7–9^** | Complete loss of p53 function, removing p53-dependent apoptosis, cell-cycle arrest, senescence, and genomic-stability maintenance | Expansion and persistence of abnormal progenitor populations, especially lymphoid cells, in the setting of impaired checkpoint and genomic-surveillance control; p53-/- splenocytes show increased S-phase entry before overt tumours | Failure of a canonical homeostatic/tumor-suppressive maintenance system comes first: loss of p53-mediated barrier function permits abnormal survival, proliferation, and genomic instability; proliferative acceleration is therefore downstream of barrier failure rather than a clean continuation of a physiological growth programme | Continued absence of p53-mediated apoptotic, senescence, and cell-cycle barriers, together with tolerance of ongoing malignant evolution, is central to tumour maintenance; p53 restoration can trigger tumour regression via apoptosis or senescence depending on tumour type | **Maintenance-insufficiency-dominant** | Best understood as failure of a central tumor-suppressive maintenance system, not as pure passive damage accumulation. The resulting pathology includes strong secondary proliferative and survival components, and p53 responses may be driven by oncogenic as well as DNA-damage-related stress, so “damage-dominant” here should not be read as a simple DNA-damage-only model. |
| **Cardiac Aging: Age-related left-ventricular hypertrophy/remodeling with diastolic dysfunction^10–12^** | Age-associated maladaptive cardiac remodeling state, with increased ventricular wall thickness/mass and reduced ventricular distensibility in the aging heart | Left-ventricular hypertrophy with impaired diastolic function, while systolic function remains preserved in the old-heart model | Remodeling/hypertrophic adaptation comes first: age-related structural remodeling, myocardial stiffness, and fibrosis drive impaired filling and diastolic dysfunction; mitochondrial, oxidative, and proteostatic abnormalities are important accompanying and downstream features rather than the clearest primary lesion in the phenotype assayed | Continued maladaptive structural remodeling and myocardial stiffening are central to maintaining the phenotype, with fibrosis, reduced compliance, and associated metabolic/proteostatic deterioration contributing to persistence of diastolic dysfunction | **Hyperfunction-dominant** | Best classified at the level of the specific old-heart phenotype assayed, not “cardiac aging” in general. Secondary mitochondrial, oxidative, and proteostatic damage are clearly present and important, but the literature provided supports structural/remodeling pathology as the dominant foregrounded process in this model. |
| **Progeroid DNA repair deficiency (Ercc1∆/-)^13,14^** | Deficiency of the ERCC1-XPF DNA repair endonuclease complex, impairing multiple DNA-repair processes and accelerating accumulation of endogenous DNA lesions | Accelerated endogenous DNA damage accumulation with transcription stress and early multi-tissue pathological decline | DNA-repair failure and lesion accumulation come first: endogenous DNA damage and genotoxic/transcription stress drive downstream senescence and progeroid tissue dysfunction | Continued unrepaired DNA damage and transcription stress, with consequent tissue-homeostasis failure and senescence burden, are central to maintaining the phenotype | **Maintenance-insufficiency-dominant** | Best understood as a canonical DNA-repair / genome-maintenance failure model, not as a one-to-one model of natural aging; it is useful here because it strongly foregrounds a damage-dominant mechanism. |
| **Bovine growth hormone transgenic mouse^15,16^** | Chronic supraphysiological GH excess with elevated GH/IGF-I signaling | Systemic overgrowth and metabolic dysregulation, including gigantism, hyperinsulinemia, and insulin resistance | Persistent growth/endocrine overdrive comes first; multi-organ pathology including glomerulosclerosis, cardiovascular disease, liver inflammation/tumors, and later oxidative stress emerges downstream with age | The pathological state is maintained in the setting of lifelong constitutive GH excess, with sustained endocrine and metabolic overdrive plausibly continuing to drive later pathology, although this maintenance dependency is inferred from the model architecture rather than directly shown by reversal experiments | **Hyperfunction-dominant** | Best treated as a mechanistically exaggerated endocrine hyperfunction model, not a direct model of wild-type aging; substantial downstream organ damage is present but appears secondary to chronic growth-axis overactivation. |

**Supplementary Table S1:** **Prospective classification of six disease models within the Aging Onion framework**. Worked application of the prospective classification schematic to each disease model discussed in the main text. For each model, the table records the initiating abnormality, earliest causal pathology, direction of causal flow, persistence requirement, resulting assignment, and major interpretive caveats. Classification was based on the proximate driver of pathology in the instantiated untreated model and was made independently of CR or rapamycin outcome

**1. Winkler, D. D. *et al.* Structural and biophysical properties of the pathogenic SOD1 variant H46R/H48Q. *Biochemistry* 48, 3436–3447 (2009).**

**2. Bhattacharya, A. *et al.* Dietary restriction but not rapamycin extends disease onset and survival of the H46R/H48Q mouse model of ALS. *Neurobiol. Aging* 33, 1829–1832 (2012).**

**3. Livi, C. B. *et al.* Rapamycin extends life span of Rb1+/− mice by inhibiting neuroendocrine tumors. *Aging* 5, 100–110 (2013).**

**4. Sharp, Z. D. Minimal effects of dietary restriction on neuroendocrine carcinogenesis in Rb+/- mice. *Carcinogenesis* 24, 179–183 (2003).**

**5. Doan, A. *et al.* RB depletion is required for the continuous growth of tumors initiated by loss of RB. *PLoS Genet.* 17, (2021).**

**6. Sherr, C. J. & McCormick, F. The RB and p53 pathways in cancer. *Cancer Cell* 2, 103–12 (2002).**

**7. Brož, D. K. & Attardi, L. D. In vivo analysis of p53 tumor suppressor function using genetically engineered mouse models. *Carcinogenesis* vol. 31 1311–1318 Preprint at https://doi.org/10.1093/carcin/bgp331 (2010).**

**8. Lozano, G. Mouse models of p53 functions. *Cold Spring Harbor perspectives in biology* vol. 2 Preprint at https://doi.org/10.1101/cshperspect.a001115 (2010).**

**9. Christy, B. *et al.* p53 and rapamycin are additive. *Oncotarget* 6, 15802–15813 (2015).**

**10. Meyer, M. *et al.* Aging diastole - root cause for atrial fibrillation and heart failure with preserved ejection fraction. *Journal of Cardiovascular Aging* vol. 4 Preprint at https://doi.org/10.20517/jca.2024.22 (2024).**

**11. Dai, D.-F., Kang, P. & Bai, H. The mTOR signaling pathway in cardiac aging. *The journal of cardiovascular aging* 3, (2023).**

**12. Dai, D. *et al.* Altered proteome turnover and remodeling by short‐term caloric restriction or rapamycin rejuvenate the aging heart. *Aging Cell* 13, 529–539 (2014).**

**13. Wong, A., Kieu, T. & Robbins, P. D. The Ercc1-/Δ mouse model of accelerated senescence and aging for identification and testing of novel senotherapeutic interventions. *Aging* 12, 24481–24483 (2020).**

**14. Birkisdóttir, M. B. *et al.* Unlike dietary restriction, rapamycin fails to extend lifespan and reduce transcription stress in progeroid DNA repair‐deficient mice. *Aging Cell* 20, (2021).**

**15. Bartke, A., Chandrashekar, V., Bailey, B., Zaczek, D. & Turyn, D. Consequences of growth hormone (GH) overexpression and GH resistance. *Neuropeptides* 36, 201–208 (2002).**

**16. Ding, J., Sackmann-Sala, L. & Kopchick, J. J. Mouse models of growth hormone action and aging: A proteomic perspective. *Proteomics* vol. 13 674–685 Preprint at https://doi.org/10.1002/pmic.201200271 (2013).**
